# Genetic Evaluation of the Nine Component Features of Hip Score in UK Labrador Retrievers

**DOI:** 10.1371/journal.pone.0013610

**Published:** 2010-10-22

**Authors:** Thomas W. Lewis, John A. Woolliams, Sarah C. Blott

**Affiliations:** 1 Kennel Club Genetics Centre at the Animal Health Trust, Animal Health Trust, Newmarket, United Kingdom; 2 The Roslin Institute and Royal (Dick) School of Veterinary Studies, University of Edinburgh, Roslin, United Kingdom; Ohio State University Medical Center, United States of America

## Abstract

The aim of this study was to explore the genetic relationship between the nine component traits comprising the British Veterinary Association (BVA) total hip score in UK registered Labrador Retrievers. Data consisted of 11,928 single records of trait scores of dogs aged between one and four years (365–1459 days) old, from radiographs evaluated between 2000 and 2007. Pedigree information was provided by the UK Kennel Club. The distribution of trait scores showed only small numbers of dogs with visible malformation in the six traits that were scored according to the severity of osteoarthritis. Linear mixed models were fitted using ASREML. Estimates of heritability ranged from 0.15 to 0.38, and litter effects from 0.04 to 0.10. Genetic correlations between all nine traits were extremely high ranging from 0.71 to 1.0, implying considerable genetic similarity. The decomposition demonstrated that aggregate scores of only the 3 traits indicative of laxity in one year old dogs was predictive of the phenotype of the remaining six scored on osteoarthritic severity in dogs at 4+ years old. The application of selection index methodology in selecting against hip dysplasia using the trait scores was explored and potential improvements in accuracy (directly related to response to selection) of over 10% are reported compared to the current total hip score. This study demonstrates that traits descriptive of joint laxity are valuable early-age predictors of osteoarthritis and shows that there is scope for improvement in the way data from the UK hip score scheme are used for selection against hip dysplasia in Labradors. This was verified via use of selection indices, which identified substantial increases in accuracy, not only via optimum coefficients, but also through an easily applicable aggregate of scores of just two or three traits only compared with the current total hip score.

## Introduction

It has been demonstrated that progress against hip dysplasia in Labradors has been discernible but slow and that the rate of genetic improvement could be greatly enhanced through selection on estimated breeding values (EBVs) rather than phenotypic hip score [1], [2], [3], [4]. However a major challenge remains: improvement in the design of recording schemes, through examination of the biological relevance of the recorded traits, to determine how response to selection against the debilitating effects of hip dysplasia may be improved.

Hip dysplasia is a developmental orthopaedic disorder characterised by the formation of a loose, ill-fitting coxofemoral (hip) joint [5]. Over time the malformation leads to abnormal wearing of bone surfaces and the appearance of the osteoarthritic signs of degenerative joint disease (DJD), such as exostosis (abnormal bone growth) and bone remodelling [6]. It has both genetic and environmental influences [1], [7], [8], [9] and is often impossible to treat since the osteoarthritis that develops is irreversible. Therefore, reduction in the prevalence of hip dysplasia through genetic selection is the best method available to provide a lasting and widespread improvement in the welfare of susceptible breeds.

The British Veterinary Association (BVA)/Kennel Club (KC) hip score scheme is in operation in the UK, EIRE, Australia and New Zealand and examines nine aspects of a pelvic radiograph for signs of malformation and secondary osteoarthritic signs of DJD in each hip. Each of the nine traits is scored and the aggregate total score is reported as an indication of hip dysplasia. However, while the scores of some traits are descriptive of deviation from normal morphology and indicative of joint laxity, others describe the degree of osteoarthritis (OA) secondary to laxity of the hip joints. Thus, the total score appears to contain elements that are both prognostic and diagnostic of OA characteristic of hip dysplasia.

The rate of participation in the BVA/KC hip score scheme in the UK is relatively good with submission of radiographs for 8–10% of all annually registered Labrador Retrievers (the most popular breed in the UK), equating to 50–60% of all dogs of that breed used for breeding. However, dogs may only be scored once in their lifetime and over ninety percent of UK registered Labrador Retrievers are scored before four years of age. Thus, it appears that many breeders wish to consider hip condition prior to breeding but that practical constraints have limited consideration to phenotypes of prospective parents only. While selection using EBVs for hip score is more accurate than using phenotypes, the total hip score as it is currently measured and aggregated may not be the most efficient phenotypic indicator of hip dysplasia for use in selection against the disease. For example, inclusion of traits relating to the pathological response to joint laxity may dilute information pertaining to the innate hip morphology. Furthermore, it may be argued that there is a moral obligation in selecting against the cause of malformation rather than the severity of the consequences.

Selection index theory provides a method of using all available phenotypic information to determine individual trait selection coefficients that will result in optimal progress towards a specific selection objective. The selection objective can be adjusted via differential weighting of some or all traits. Although selection indices have most often been used in livestock breeding (where weights have been economically derived) it may be achievable to develop ‘welfare’ values and construct an index for optimal selection against inherited disease, of which canine hip dysplasia is a prime example. For the nine traits currently measured under the BVA/KC scheme this would involve: 1) being clear on the extent to which traits are definitive of disease and which are biomarkers or secondary consequences, 2) analysing the genetic contribution to prediction of disease, and 3) deriving how the traits may be best weighted to provide the most accurate predictions. Such an undertaking would reveal the selection coefficients of traits producing the maximum progress against hip dysplasia, but would require extensive debate on the precise calculation of ‘welfare values’ for each of the nine traits.

This study has the objectives of examining 1) the genetic parameters of all nine traits in the BVA/KC hip scoring scheme in 1–4 year old dogs using mixed model analyses, 2) the predictive ability of traits describing joint laxity and secondary osteoarthritic signs using EBVs, and 3) the advantages conferred by a selection index in the effective selection against inherited disease.

## Results

### Distributions of score for the nine traits

**Figure 1:**
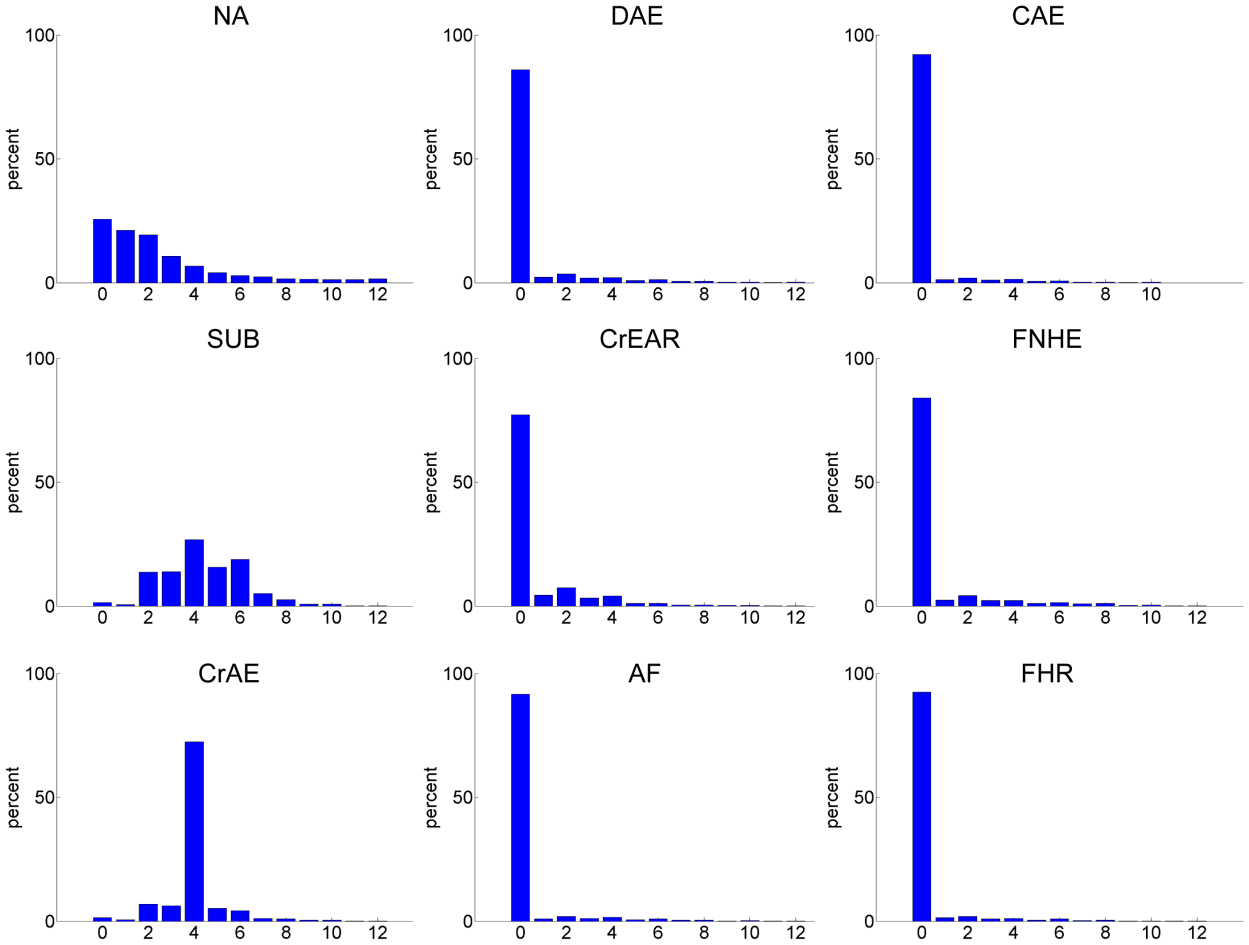
Distribution of total score. Left + right hip score for the 9 traits contributing to total hip score.

The distribution of scores for each of the nine traits (1 = Norberg Angle, NA; 2 = Subluxation, SUB; 3 = Cranial Acetabular Edge, CrAE; 4 = Dorsal Acetabular Edge, DAE; 5 = Cranial Effective Acetabular Rim, CrEAR; 6 = Acetabular Fossa, AF; 7 = Caudal Acetabular Edge, CAE; 8 = Femoral Head and Neck Exostosis, FHNE; and 9 = Femoral Head Recontouring, FHR) are shown in Figure 1 and statistics in Table 1. Over 91% of all records for AF, CAE, and FHR had been scored zero and 86%, 84% and 77% of all records were scored zero for DAE, FHNE and CrEAR respectively. Thus, the means of these six traits are smaller and coefficients of skew larger than for NA, SUB and CrAE (Table 1), with the important implication that there is very little phenotypic deviation from ‘normal’ morphology in these six features of the hip joint in UK registered Labradors evaluated between 1 and 4 years of age. The distribution of CrAE has a modal score of four (72% of records), rather than zero as was the case for traits 4–9, affording a lower coefficient of skew and a higher mean, but a standard deviation of comparable magnitude to that for traits 4–9. NA and SUB are more evenly distributed over scores and consequently showed greater phenotypic variation. The distribution of NA scores was positively skewed, reflecting the categorisation of abnormality in what would be expected to be a normally distributed, empirically measured trait.

**Table 1:** Summary statistics for the 9 traits contributing to total hip score.

Component		Mean	S.D.	CV	Skew	Correlation L&R
1	NA	2.436	2.731	1.121	1.646	0.619
2	SUB	4.389	1.761	0.401	0.306	0.447
3	CrAE	3.984	1.193	0.299	1.002	0.757
4	DAE	0.547	1.692	3.093	3.856	0.768
5	CrEAR	0.692	1.570	2.269	2.862	0.799
6	AF	0.340	1.338	3.935	4.754	0.780
7	CAE	0.281	1.150	4.093	4.954	0.752
8	FHNE	0.610	1.712	2.807	3.335	0.755
9	FHR	0.266	1.119	4.207	5.108	0.760

Mean, standard deviation (S.D.), coefficient of variation (CV), coefficient of skew, and phenotypic correlation between left and right score for each of the 9 traits that sum to the total hip score according to the BVA/KC scheme (NA  =  Norberg angle, SUB  =  subluxation, CrAE  =  cranial acetabular edge, DAE  =  dorsal acetabular edge, CrEAR  =  cranial effective acetabular rim, AF  =  acetabular fossa, CAE  =  caudal acetabular edge, FHNE  =  femoral head and neck exostosis, FHR  =  femoral head recontouring).

### Heritabilities and genetic correlations of the nine features of total hip score

An objective of this study was to estimate the genetic parameters of the nine traits and explore the genetic relationships between them. Estimates of heritability and litter variation of untransformed scores from univariate analysis are presented in Table 2. Heritability estimates were highest for SUB (0.38±0.026) and NA (0.37±0.027), with estimates for all seven remaining traits ranging from 0.15 (CAE and AF) to 0.24 (FHNE). The inclusion of litter in the model was significant (P<0.05) for all traits except FHR (P<0.10 using likelihood ratio test). For all traits the variation due to litter explained a much smaller fraction of total variation than the genetic variation, ranging from 0.04 (CAE) to 0.10 (SUB).

**Table 2:** Variance components of hip score traits.

Component		Heritability (s.e.)		Litter effect (s.e.)	
1	NA	0.37	0.027	0.08	0.017
2	SUB	0.38	0.026	0.10	0.017
3	CrAE	0.21	0.023	0.06	0.017
4	DAE	0.18	0.024	0.06	0.018
5	CrEAR	0.21	0.024	0.09	0.018
6	AF	0.15	0.023	0.08	0.019
7	CAE	0.15	0.022	0.04	0.018
8	FHNE	0.24	0.026	0.07	0.018
9	FHR	0.19	0.025	0.04	0.019

Estimates of heritability and the fraction of phenotypic variance explained by litter on each of the nine traits that sum to the total hip score according to the BVA/KC scheme (NA  =  Norberg angle, SUB  =  subluxation, CrAE  =  cranial acetabular edge, DAE  =  dorsal acetabular edge, CrEAR  =  cranial effective acetabular rim, AF  =  acetabular fossa, CAE  =  caudal acetabular edge, FHNE  =  femoral head and neck exostosis, FHR  =  femoral head recontouring).

The genetic and phenotypic correlations between each pair of the nine traits (and standard errors in parentheses) are presented in Table 3. The genetic correlations range from 0.71 between SUB and FHR, up to 1.0 between DAE and AF, and FHR and AF. Overall, the genetic correlations were extremely high, with only three correlations at less than 0.8 (SUB with each of AF, CAE and FHR). The phenotypic correlations followed a similar pattern (ranging from 0.47 between SUB and FHR, to 0.88 between AF and CAE) but were lower in magnitude than the genetic correlations. Residual or environmental correlations showed a much wider range than the genetic correlations (from 0.37 between SUB and FHR, to 0.86 between AF and CAE) reflecting more differential environmental influences among traits.

**Table 3:** Genetic and phenotypic correlations among traits contributing to total hip score.

	NA	SUB	CrAE	DAE	CrEAR	AF	CAE	FHNE	FHR
NA		0.75 (0.005)	0.66 (0.007)	0.71 (0.005)	0.74 (0.005)	0.63 (0.006)	0.60 (0.006)	0.70 (0.005)	0.58 (0.006)
SUB	0.85 (0.018)		0.66 (0.006)	0.57 (0.007)	0.61 (0.006)	0.51 (0.007)	0.48 (0.008)	0.57 (0.007)	0.47 (0.008)
CrAE	0.93 (0.018)	0.93 (0.018)		0.70 (0.005)	0.70 (0.005)	0.65 (0.006)	0.62 (0.006)	0.65 (0.006)	0.61 (0.006)
DAE	0.96 (0.019)	0.83 (0.033)	0.93 (0.022)		0.84 (0.002)	0.87 (0.002)	0.85 (0.003)	0.85 (0.003)	0.82 (0.003)
CrEAR	0.93 (0.017)	0.86 (0.026)	0.95 (0.016)	0.97 (0.011)		0.77 (0.004)	0.73 (0.004)	0.80 (0.004)	0.71 (0.005)
AF	0.90 (0.037)	0.78 (0.044)	0.89 (0.033)	1.00 (0.000)	0.91 (0.025)		0.88 (0.002)	0.83 (0.003)	0.84 (0.003)
CAE	0.91 (0.033)	0.78 (0.043)	0.89 (0.032)	0.97 (0.013)	0.93 (0.022)	0.99 (0.007)		0.82 (0.003)	0.83 (0.003)
FHNE	0.84 (0.027)	0.81 (0.032)	0.90 (0.026)	0.99 (0.006)	0.95 (0.014)	0.97 (0.012)	0.95 (0.014)		0.86 (0.003)
FHR	0.83 (0.040)	0.71 (0.046)	0.87 (0.034)	0.99 (0.011)	0.90 (0.028)	1.00 (0.006)	0.95 (0.016)	0.96 (0.013)	

Genetic (below diagonal) and phenotypic (above diagonal) correlations between each of the nine traits that sum to the total hip score according to the BVA/KC scheme (NA  =  Norberg angle, SUB  =  subluxation, CrAE  =  cranial acetabular edge, DAE  =  dorsal acetabular edge, CrEAR  =  cranial effective acetabular rim, AF  =  acetabular fossa, CAE  =  caudal acetabular edge, FHNE  =  femoral head and neck exostosis, FHR  =  femoral head recontouring). Standard errors in parentheses.

### Genetic correlations between groups of aggregate scores

The relationship between groups of traits according to scoring criteria was subsequently explored. The genetic correlation between SCORE_4–9_ (aggregate score of traits scored on the pathological signs of OA) and SCORE_1–3_ (aggregate score of traits scored largely on signs of morphological malformation) was 0.89 (±0.023) when both were transformed using natural logarithm of aggregate score +1, and 0.92 (±0.020) when untransformed, indicating substantial genetic similarity between the two groups. Such high genetic correlations indicate that morphology will potentially act as a good predictor of damage due to OA. However on both scales genetic correlations were significantly smaller than one (P<0.001), implying that whilst SCORE_1–3_ and SCORE_4–9_ are genetically very similar traits, they are not genetically identical on these scales. The heritability estimates of SCORE_1–3_ were 0.38 (±0.026) and 0.33 0(±0.025), and of SCORE_4–9_ were 0.18 (±0.022) and 0.25 (±0.025), when untransformed and transformed respectively.

### Prediction of SCORE_4–9_ using SCORE_1–3_

Having ascertained the magnitude of the genetic relationship between these two groups of traits, the predictive ability of morphological traits on the pathologically descriptive traits was assessed using EBVs calculated from dogs scored at a young age and using them as predictors of phenotypes of offspring scored at a late age (see Materials and Methods). The correlations of ‘early age’ EBVs for SCORE_1–3_, SCORE_4–9_ and total score (SCORE_1–9_) with ‘late age’ phenotypes are shown in Table 4. All correlations were significantly greater than zero, except that for EBV_4–9_ with SCORE_1–3_. EBV_1–3_ and EBV_1–9_ were each similarly correlated with the three score groups, but in each case the correlation with EBV_1–3_ was of greater magnitude representing greater predictive ability. The maximum correlation was of early score EBV_1–3_ on SCORE_1–9_ at 4+ years old (r = 0.22). Correlations of EBV_1–3_ and EBV_1–9_ with each score group were significantly higher than EBV_4–9_ for both SCORE_1–9_ and SCORE_4–9_. Thus, EBV_1–3_ calculated at birth using data from one year old dogs is a more accurate predictor of subsequent SCORE_4–9_ at 4+ years old than the EBV_4–9_ of the same trait. Therefore selection on EBVs for SCORE_1–3_ using data from young dogs will have no detrimental impact on the improvement of SCORE_4–9_, and may rather be slightly more accurate than EBVs for total score at improving all component traits.

**Table 4:** Correlation of EBVs and phenotypes for 3 groupings of hip score traits.

	SCORE1–9	SCORE1–3	SCORE4–9
EBV_1–9_	0.2086 (0.0003)	0.1958 (0.0006)	0.1811 (0.0018)
EBV_1–3_	0.2165 (0.0002)	0.2065 (0.0003)	0.1831 (0.0014)
EBV_4–9_	0.1276 (0.0272)	0.1024 (0.0765)	0.1453 (0.0117)

Correlations of ‘early age scored’ EBVs for SCORE1–3, SCORE4–9 and SCORE1–9 with phenotypes in 300 ‘late age’ progeny at time of scoring. Standard errors in parentheses.

### Demonstration of suitability of a selection index for hip dysplasia

An alternative approach to explore the potential improvements in response to selection is to combine them into an index *I* with ‘index coefficients’ for each of the traits 1–9 that are no longer constrained to 1 or 0. The coefficients are derived to provide the highest accuracy for an objective *H* which places relative values on the progress achieved in each of the traits. This is explained in more detail in Materials and Methods. The accuracies of optimised selection coefficients were, as expected, always highest when information on all nine traits was incorporated into the index *I*, whatever the selection objective (*H*). When all nine traits in *H* were equally valued after being scaled by their standardised phenotypic variation (**a**_1_), the selection coefficients were: 0.538 (NA), 0.546 (SUB), 0.121 (CrAE), −0.184 (DAE), −0.126 (CrEAR), −0.310 (AF), −0.186 (CAE), 0.371 (FHNE), 0.564 (FHR). This profile of index weights is quantitatively similar for the two other objective scenarios that were considered, the total score as calculated currently by BVA/KC (**a**_2_) or relative values based upon the impact of OA (**a**_3_). On a technical note, the negative values in the index coefficients do *not* imply that the index results in selection for features definitive of dysplasia, but rather signifies that due to the high phenotypic and genetic correlations some traits act as ‘environmental corrections’ to more informative traits that are richer in genetic information, allowing better prediction of genetic merit.

Table 5 displays the accuracies (directly related to rate of improvement) of the optimum selection coefficients determined by selection index theory for each of the three objectives considered, together with accuracies obtained by using index coefficients representing simplified aggregate scores of the nine traits. The results show that using aggregate total score is only between 88–90% as accurate as the optimum coefficients over all 3 scenarios for the relative values in the objective. Furthermore, selection with indices representative of aggregate scores of morphological traits (NA+SUB+CrAE and NA+SUB) were between 10–12% more accurate than total score for **a**_1_ and **a**_2_ and 6–7% more accurate for **a**_3_ where higher value is placed on improving the traits that are descriptive of pathological OA.

**Table 5:** Accuracies of selection coefficients for optimum and derived indices over different welfare weights.

Weights (a)	Selection coefficients (b)				
	Optimum	[111111111]^T^	[111000000]^T^	[110000000]^T^	[000111111]^T^
1/σ_Pi_	0.616	0.545	0.598	0.602	0.456
[111111111]^T^	0.619	0.545	0.603	0.608	0.454
[00½111111]^T^	0.595	0.537	0.570	0.573	0.462

Accuracies from selection indices with different coefficients (***b***) (from left to right: the optimum, aggregate total hip score, aggregate NA+SUB+CrAE, aggregate NA+SUB, aggregate DAE+CrEAR+AF+CAE+FHNE+FHR), and at different welfare weights (***a***) (from top to bottom: scaled and equal, unscaled and equal, and weighted on impact on OA).

## Discussion

The results from this study suggest that there is significant scope for improvement in the application of data from the UK hip score scheme to selection against hip dysplasia in Labradors. The aggregate score of just three of the trait scores that sum to the total hip score was demonstrated to be both substantially genetically related to, and adequately predictive of, the remaining six, as well as exhibiting marked variation in the malformation indicative of joint laxity at 1–4 years old. The results from analyses with selection indices further underline the genetic predictive value of the morphological traits on the pathology associated with hip dysplasia, with optimum selection coefficients yielding a 14% increase in accuracy (directly related to response to selection) over the current aggregate score at improving all nine traits. Furthermore an easily applicable aggregate of scores of morphological traits would only be marginally less accurate than the optimum.

The six traits indicative of pathology DAE, CrEAR, AF, CAE, FHNE and FHR showed no malformation in the vast majority of data from 1–4 year old dogs, the age at which >90% of dogs are scored. All these traits are graded on the degree of pathological OA in response to joint laxity and were subject to detrimental age affects. Conversely, NA, SUB and CrAE were scored >0 for the majority of dogs, indicative of sub-clinical malformation detectable at an early age and implying that the causal factors are effective at one year old. It therefore appears that in the many dogs displaying malformation in these three traits (for example 96% of animals had a bilateral SUB score of 2–8) either the development of osteoarthritic signs is rare; or more likely that in the large majority of cases insufficient time had passed at age of evaluation for such signs to develop. Breeders wishing to take hip score into account in their selections inevitably score dogs prior to breeding age; an age at which the pathological responses to joint laxity appear not to have had sufficient time to become manifest in all but the most extreme cases.

The heritability estimates of NA and SUB are substantially higher than those for the remaining seven traits, indicating that these traits concerning (near) innate morphology are richer in genetic information than those traits describing development and severity of OA. Coupled with the high genetic correlations of NA and SUB with traits indicative of the pathological signs associated with dysplasia this makes them of high value for selection against hip dysplasia. The heritability of NA could potentially be further increased if the angle measured was reported since categorisation simply adds measurement error and serves no predictive purpose. Whilst it has been suggested that differences in heritability estimates reflect the ease of categorisation according to the scoring criteria and therefore smaller diagnostic variation [10], an additional reason may be due to the low prevalence of abnormalities in traits 4–9 at the age of scoring. The scoring of these traits is dominated by the binary categorisation of ‘normal’ and ‘abnormal’ and for such binary traits in a liability model, heritability will increase with prevalence. One consequence of this is that as the prevalence increases with age the heritabilities may become higher as they begin to express a more complete spectrum of liabilities, however this is of only academic interest since delay in hip scoring will drastically reduce the genetic progress through fewer prospective parents scored by breeding age and/or longer generation intervals. Nevertheless, these six traits do appear heritable to a modest degree (0.15–0.24) in 1–4 year old dogs suggesting they can contribute useful genetic information on the pathological response to joint laxity, although the magnitude of the heritabilities limit their usefulness.

The large and positive genetic correlations between the traits are advantageous, indicating that selection for improvement in any one trait would result in improvement in them all. However, because scoring of six of the traits is dependent on osteoarthritic signs, they may act as repeat indicators of the severity of the pathological response to joint laxity. This may partly explain some of the extremely high genetic correlations, particularly between DAE, CrEAR, AF, CAE and FHNE, all of which are graded according to the severity of exostosis observed. Furthermore, DAE, CrEAR, AF, CAE, FHNE and FHR appear to be conditional on NA, SUB and CrAE. For example it is very unlikely that dogs with an aggregate score of zero for NA, SUB and CrAE would have high scores for the other six traits. Therefore selection for improvement in NA, SUB and CrAE will improve the other trait scores partly by easing the predicate. However, although large the genetic correlation between the two groups of traits was significantly less than one indicating non-identicality, possibly due to genetic variation in the pathological response to laxity of the coxofemoral joint. Selection for improvement in DAE, CrEAR, AF, CAE, FHNE and FHR would therefore not only result in less efficient selection against the predictive traits (NA, SUB and CrAE) but a reduction in propensity to display a biologically normal pathological response to laxity as well. Similarly, while the traits examined are one step away from the clinical manifestation of hip dysplasia as pain and lameness, selection against the symptom risks the response being a reduction in normal expression of such symptom, even when the underlying cause remains.

This study has also demonstrated that EBVs of NA, SUB and CrAE derived from scoring of one year old dogs are better predictors of OA in the other six traits in later life than are EBVs for these six traits themselves. This result strengthens the case of causality of NA, SUB and CrAE on the other six traits, implied by the scoring criteria and supported by the high genetic correlations. Although this might lead to the proposal of simply dropping traits 4–9 from consideration in selection against hip dysplasia, results from the selection index demonstrated that the inclusion of all information always results in higher accuracy of selection coefficients, no matter what the selection objective. Thus their continued presence in the BVA/KC scheme is justified. The presence of negative coefficients for four of the traits descriptive of pathological OA indicates that they serve a purpose as ‘environmental corrections’. To give a simple illustration, consider two traits, and assume merit is positively related to each trait: the first has heritability 0.5, with a phenotype obtained by summing two numbers drawn from N(0,1), the first representing the breeding value, the second representing the environmental deviation; the second trait consists of only the second number, i.e. environmental deviation. It is clear that the optimum predictor of genetic merit is the first trait minus the second, even though both traits increase as merit increases. The second trait which is richer in environmental variation is acting to correct the first so as to better predict genetic merit. The same phenomena will occur with the nine traits here but the relationships are obviously more complex.

The results from analyses of selection indices have not only quantitatively endorsed the conclusions of the predictive ability of the morphological traits on those describing pathology, but have also quantified the improvement achievable by use of either optimum coefficients derived from index theory, or aggregate scores of traits describing hip morphology only. Such improvement initially seems counter-intuitive since it appears that information is being discarded, but in fact may be explained due to the relative increase in environmental variation that occurs when the scores of pathology traits are included. Heuristically, the index ***b*** =  [111000000]^T^ is ‘closer’ to the optimum coefficients than the total score (***b*** =  [111111111]^T^). Results from this study indicate that improvements in selective efficacy of >10% are available simply by re-weighting the information already collected.

The study described decomposes of the genetic and phenotypic variances of the current hip score as observed into it the components arising from the 9 traits contributing to it, although this brings with it difficulties of distribution with ordinal data, most notably in traits 4–9. For these traits we have maintained the differentiation among positive scores with the supposition that the use of the scale by the BVA/KC panel of experts embodies confidence in diagnosis as well as degree and that ignoring scoring categories may ‘coarsen’ the scale of analysis. Nevertheless, we replicated the analyses described and calculated optimum selection coefficients considering traits 4–9 as scaled binomial traits (0 = unaffected, *a*>0 = affected), where *a* is chosen to maintain the same mean value, but the changes in optimum selection coefficients from those reported were negligible. Furthermore, the objectives of this study rely on a seamless framework that encompasses the individual trait scores and their total sum, which is the current reported total hip score. Clarity of results for ‘end users’ relies in part on the maintenance of the framework of linearity; otherwise the transformed sum is no longer the sum of the transformed. Further analysis of individual trait transformation to address distributional issues more directly might be more advantageous once the total hip score is no longer seen as the widespread and accepted evaluation of hip condition, for example once EBVs are publically available.

The demonstration of how selection index theory can improve the efficacy of selection against hip dysplasia has far reaching implications illustrating that substantial increases in response to selection are easily achievable. Such methods could be extended to breeding for health in breeds where there are many inherited diseases. The principles involved are easily transferable from their traditional use in livestock production, although the economic values must be replaced with values derived from the welfare impact of each disease or feature of disease. Hip dysplasia is probably unique among companion animals in the provision of such a large quantity of data on so many of the features of disease, but the disease and the screening data is complex, reflecting underlying genetics, environmental effects and the debilitating developmental consequences of innate malformation. Although more detailed exploration of the assignment of welfare values and investigation into the effect of transformation of scores of the features considered to describe hip dysplasia is desirable, as the one of the most prominent canine inherited diseases the increases in selective accuracy identified in this study emphasize easily applicable changes to the ways data is used that will result in a greater response to selection.

## Materials and Methods

### Data

**Figure 2:**
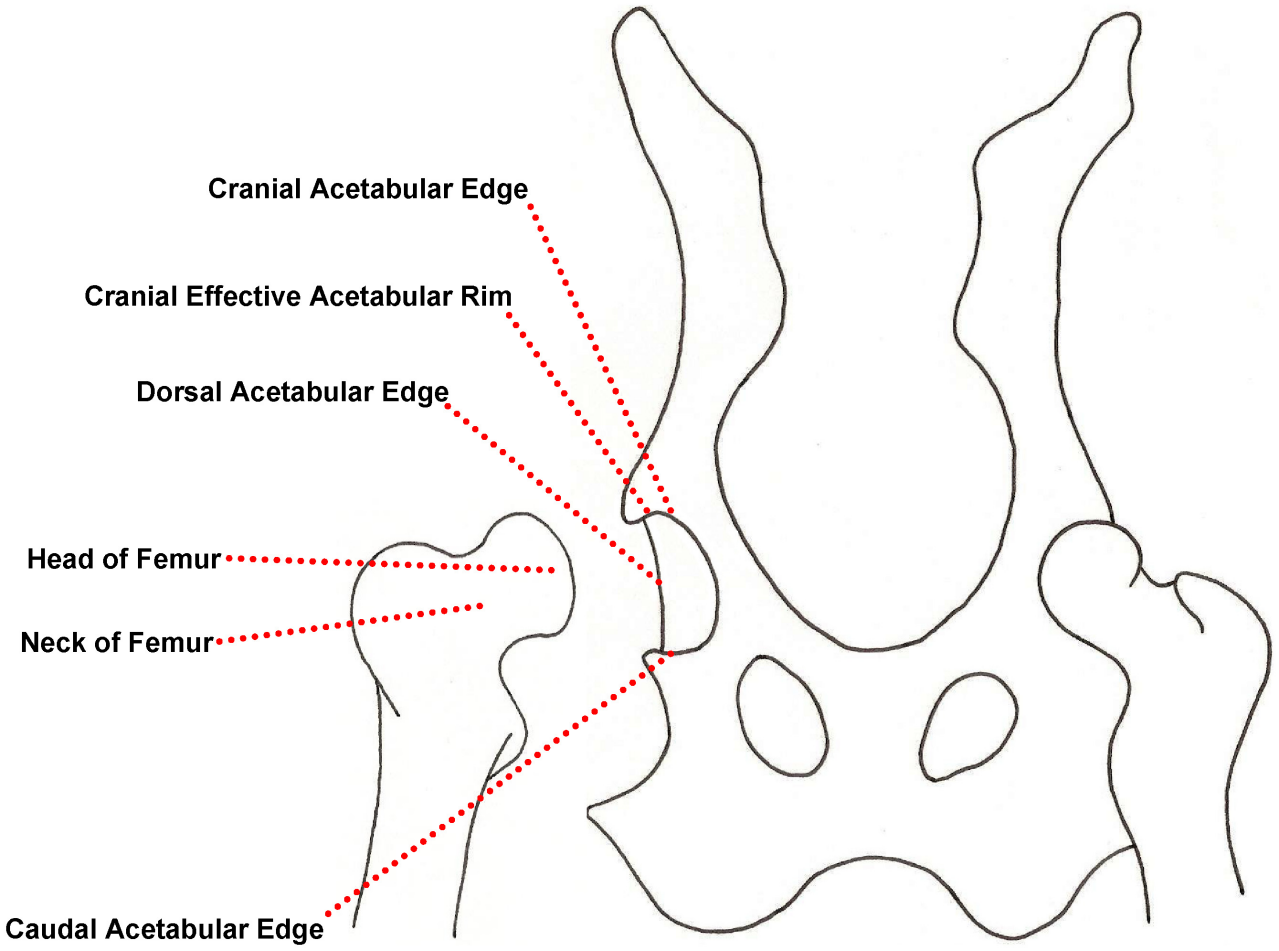
Diagrammatical representation of the skeletal features of the hip joint. Features pertinent to those graded by the BVA/KC hip score scheme.

In the BVA/KC hip score scheme, radiographs of the pelvic area in the “extended hip” position are taken by a general practice vet according to standardised protocols and are voluntarily submitted to the BVA for evaluation by three members of a panel of certified radiologists or small animal surgeons. The anatomical features of the canine pelvis involved in evaluation are represented in Figure 2 and a précis of scoring criteria for the nine features that comprise the total hip score are given in supplementary material (File S1 and Figure S1). Score data on the nine traits of both (left and right) hips was generated by the BVA/KC scheme (computerized records kindly provided by Dr Malcolm Willis). 11,928 records of dogs with individual trait scores directly corresponded to records in the larger data set described in a previous study [11], which was limited to animals ≥1 and <4 years old (365–1459 days inclusive) at the time of radiograph, and evaluated between 2000 and 2007. Initial analysis utilised these data, and feature scores of left and right hips were summed to give a total score for each of the nine traits. The data were in similar proportions according to class of sex and coat colour as those previously reported [11]. Records were distributed over years of evaluation as shown in table 6.

**Table 6:** Distribution of data over year of evaluation.

Evaluation year	Count	Percent
2000	1963	16.46%
2001	1804	15.12%
2002	2039	17.09%
2003	2483	20.82%
2004	2543	21.32%
2005	1035	8.68%
2006	61	0.51%

The pedigree used in the analyses was identical to the one described previously [11], unless stated otherwise.

### Statistical Analysis

Statistical analyses of the data had the objectives of fitting mixed linear models using ASREML [12] to estimate the genetic parameters of the nine traits. It was previously ascertained that a logarithmic transformation was appropriate given the skewed nature of the total hip score [11]. However score distribution was not consistent across the nine traits and analysis of one trait (SUB) with the family of power transformations [13] determined that in this case logarithm was not the optimal scale for analysis. Furthermore, aggregate total score relates to a simple, equal weighting of trait scores and maintaining this format would simplify evaluation with selection index models. Therefore analyses of the nine individual traits were conducted on untransformed scores.

Substantial genetic similarity in log-transformed aggregate score of left and right hips, with near perfect genetic correlation and near identical genetic and environmental variances was reported previously [11], inferring that a sum of scores of left and right hips was a reasonable simplification. Furthermore, preliminary investigations of this study determined that the genetic correlation between the untransformed scores of left and right hips for two of the nine traits was very close to one (0.996±0.007 for CrAE and 0.998±0.006 for FHNE) and therefore the phenotypes used in all analyses were sums of scores from both hips for each of the nine traits.

Small but significant unique litter effect on total hip score were previously reported [11], having failed to detect significant maternal and litter specific effects, and so litter effects only were included in this analysis.

Initially, nine univariate analyses were conducted (one for each trait) to determine variance components. Subsequently, thirty six (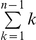, where n = 9) pair-wise bivariate analyses were conducted to estimate the genetic correlations between every pair of the nine traits comprising the BVA/KC total hip score. The general form of the linear model was as follows:
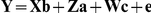
where **Y** is the vector of observations; **X**, **W** and **Z** are known incidence matrices, **b** is the vector of fixed effects, **a** is the vector of random additive genetic effects with the distribution assumed to be multivariate normal (MVN), with parameters (0, σ^2^_a_ **A**); **c** is the vector of random litter effects with the distribution assumed to be MVN, with parameters (0, σ^2^_c_ **I**); and **e** is the vector of residuals distributed MVN with parameters (0, σ^2^_e_ **I**); and where **I** denotes an identity matrix of the appropriate size, **A** is the numerator relationship matrix, and σ^2^ is a scalar denoting variance. In the case of bivariate analyses σ^2^ was replaced by the appropriate covariance matrix for traits, **Σ**, with the direct product operator. The fixed effects included in the model were: sex, age, season of birth (winter  =  January – March, spring  =  April – June, summer  =  July – September, autumn  =  October – December) and year of evaluation. Age in days was centred and scaled according to the mean and SD of the data used (for reference, mean  = 709.60, SD = 291.10 for data currently described; n = 11,928) and a polynomial regression was fitted. These fixed effects are a simpler form to those fitted previously [11], and therefore more tractable in a computationally more intensive series of models. However they are adequate to describe the effects on score.

### Component score group relationships

The genetic correlation between two groups of traits was calculated to determine the likely effect that selection on fewer traits would have on the others. Scores for NA, SUB and CrAE (SCORE_1–3_) were summed (out of a maximum of 36) since these features are scored largely on the detection of morphological malformation (i.e. independent of OA). Scores for DAE, CrEAR, AF, CAE, FHNE and FHR (SCORE_4–9_) were summed (with a maximum of 70) since the scoring of these traits is entirely dependent on the severity of observed pathological malformation (i.e. signs of OA). Pairwise bivariate analyses were conducted using 1) the raw scores and 2) log_e_(score+1) transformation of each of the aggregate groups (SCORE_4–9_ and SCORE_1–3_), as described previously. This logarithmic transformation was shown to normalise the skewed distribution of aggregate total hip score [11].

### Predictive power of groups of component scores

The predictive power of EBVs derived from SCORE_1–3_, SCORE_4–9_ and total score (SCORE_1–9_) on each of the phenotypic groups of scores was assessed. Data from a cohort of dogs scored at one year (365–729 days) old (n = 3,912) and born before 1995 were isolated. The data were restricted to records where sex was explicitly stated as male or female, coat colour stated as one of the 3 ‘permitted’ colours (black, chocolate and yellow) and scores were within the prescribed ranges. A linear mixed model was fitted as previously described to calculate EBVs for log_e_(1+ SCORE_1–3_), log_e_(1+ SCORE_4–9_) and log_e_(1+ SCORE_1–9_) of all animals with data and in the pedigree. The fixed effects included in the model were as previously described. Pedigree of up to a maximum of four generations of ancestors of dogs with data was used in the analysis (n = 30,527). The pedigree was augmented with the 300 progeny of animals with ‘early score’ data that were themselves ‘late scored’ at over 4 years (1460+ days) old and born after 1995, in order to obtain ‘early score’ EBVs for dogs with a ‘late score’ phenotype. Correlation of EBV_1–3_, EBV_4–9_ and EBV_1–9_ with transformed phenotypes SCORE_1–3_, SCORE_4–9_ and SCORE_1–9_ in the 300 dogs scored at >4 years old illustrated the predictive power of traits in 1 year old dogs on traits in older dogs (>4 years old) when the clinical signs of joint disease have had more time to become manifest.

### Demonstration of a selection index for hip dysplasia

The construction of a selection index was undertaken to demonstrate simple improvements in selective efficacy that are available via the use of optimally weighted EBVs of the BVA/KC hip score data. A more detailed description of the methodology of selection indices is given by Cameron [14]. Optimum selection indices for selection objectives (*H*) using selection criteria (*I*) were obtained with coefficients (*b*) calculated:
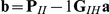
where **P** is the phenotypic variance/covariance matrix and **G** is the additive genetic covariance matrix derived from the individual pairwise bivariate analysis of each of the 9 traits scored. **G** was made positive definite by the substitution of two small, negative eigenvalues by small, positive values; this correction amounted to a change of 0.4% of the sum of the magnitudes of the original eigenvalues. This procedure minimises the Frobenius distance of the corrected matrix from the original estimate. Subscripts *I* and *H* define the relevant sub-matrices of **P** and **G** for traits in *I* and *H*. **a** represents the vector of relative values for traits in the selection objective (*H*) defining the aggregate breeding value and may be adjusted to reflect the welfare values implicit in each scored trait. Welfare values would need to be derived from consideration of the impact on welfare of malformation of each trait considered. In this study, optimum indices were considered using: 1) **a** = 1/σ_Pi_ for trait *i*, attaching equal welfare value to the traits when standardised phenotypically; 2) **a** =  [1,1,1,1,1,1,1,1,1]^T^, representing an equal welfare value of all nine traits and therefore aggregate breeding value equal to breeding value for the total hip score; 3) **a** =  [0, 0, ½, 1, 1, 1, 1, 1, 1]^ T^ where the aggregate breeding value is a measure of propensity for pathological damage, which might be considered to better reflect welfare. For each value of **a**, the accuracy of the optimum index was compared to: 1) current selection practice which uses the total hip score, as represented by **b** ^T^ =  [1 1 1 1 1 1 1 1 1]; 2) the aggregate score of traits scored wholly or partially on morphological malformation only, represented by **b** ^T^ =  [1 1 1 0 0 0 0 0 0] (NA+SUB+CrAE) or **b** ^T^ =  [1 1 0 0 0 0 0 0 0] (NA+SUB); and 3) the aggregate score of traits scored wholly on pathological malformation, represented by **b** ^T^ =  [0 0 0 1 1 1 1 1 1] (DAE+CrEAR+AF+CAE+FHNE+FHR). The accuracy of the selection index was determined as the correlation of the aggregate breeding value with the index value.

## Supporting Information

File S1 A précis of scoring criteria for the nine features that comprise the total hip score.
(0.03 MB DOC)

Figure S1 Three examples of progressively deteriorating Norberg Angle. The left is a radiograph of a hip joint showing a positive angle indicating good acetabular depth. The middle radiograph is an example of a small negative Norberg Angle, and the right radiograph an example of a large negative angle. Other signs of joint malformation and osteoarthritic effects may also be seen. Images courtesy of Ruth Dennis.
(8.17 MB TIF)
