# Supplementary material for: Genetic Evaluation of the Nine Component Features of Hip Score in UK Labrador Retrievers
Source: PLoS One. 2010 Oct 22;5(10):e13610. doi: 10.1371/journal.pone.0013610 (PMC2962649; doi:10.1371/journal.pone.0013610)
Supplement: File S1 — A précis of scoring criteria for the nine features that comprise the total hip score. (0.03 MB DOC) [file pone.0013610.s001.doc]

File S1 – Scoring criteria for the nine features comprising BVA/KC total hip score.

A brief description of the nine component features comprising the total hip score and broad scoring criteria under the BVA/KC scheme follows adapted from Gibbs [10].

1) The Norberg angle (NA) encapsulates the congruence of the head of the femur and the acetabulum, and the length of the cranial acetabular edge (CrAE). Together these give an indication of the depth of the acetabulum. The angle measured is that from the perpendicular of a line between the two centres of the femoral heads, to the extent of the CrEAR (Figure S2). It is the only component feature requiring empirical measurement but is then categorised to give the a score from zero to six for each hip; an angle of +15º and over graded 0, +10 º to +14 º graded 1, +5 º to +9 º graded 2, 0 º to +4 º graded 3, -1 º to -5 º graded 4, -6 º to -10 º graded 5, and -11 º or less graded 6. Scores for each hip are summed, giving a total score of NA from 0 to 12.

2) The degree of subluxation (SUB) is evaluated by the ‘fit’ of the femoral head into the acetabulum as judged by the positions of the femoral head centres and the dorsal acetabular edge (DAE) and the regularity and consistency of the narrow joint space (particularly in lower scoring hips). SUB is scored from zero to 6 for each hip (zero to 12 in total). A score of 2 denotes the centre of the femoral head lies superimposed on the DAE, of 3 indicates that less than ½ but more than ¼ of the femoral head is lying medial to the DAE, and increasing severity culminates in a score of 6 representing complete dislocation.

3) The scoring of CrAE quantifies deviations in the shape and contour of the CrAE from a zero score where the CrAE is convex and uniformly curved to match the contour of the adjacent femoral head. CAE is scored from zero to 6 for each hip with deviations from the uniform curve matching the contour of the femoral head result in a higher score. Flattening of the outer quarter of the CrAE with divergence of the joint space results in a score of 1 or 2. Scores of 3 and above are awarded when there is evidence of abnormal bone wearing or remodelling of the CrAE. Scores for either hip are summed to give a total out of 12.

4) The DAE is normally slightly curved (score of zero) and a score of 1 is assigned due to a change in contour. Scores of 2 and above are explicitly related to evidence of pathological changes due to osteoarthritis, most commonly exostosis (abnormal bone growth). Maximum score per hip is 6, and scores are summed to a total out of 12.

5) Cranial effective acetabular rim (CrEAR) is scored on the degree of detectable abnormality in the form of either exostosis or in a ‘rounding’ of the junction between the CrAE and DAE, often as a result of wearing. CrEAR is scored from zero to 6 for either hip and zero to12 in total.

6) The acetabular fossa (AF) [*fossa* refers to a depression in bone which lodges another structure] is acknowledged by Gibbs [10] as difficult to score by radiograph. Bone deposition detected by radiograph is usually associated with subluxation and the author implies that scoring of the AF is somewhat inaccurate but highly correlated with other features. AF is scored from zero to 6 for each hip and summed to a total out of 12.

7) The caudal acetabular edge (CAE) is scored on changes from a clean line, mainly due to exostosis with signs of wear being present in advanced cases. CAE is scored out of 5 for each hip, and so from zero to 10 in total. notes that CAE is the feature of the acetabulum subject to the widest range of radiographical variation, dependent to some extent on the positioning of the animal when x-rayed.

8) The grading of femoral head and neck exostosis (FHNE) is dependent, as its name suggests, on the degree of exostosis observed. FHNE is scored from zero to 6 for each hip and summed to a total out of 12.

9) The feature ‘femoral head recontouring’ (FHR) evaluates the extent to which the femoral head is affected by instability in the hip joint through remodelling from new bone formation and loss of subchondral bone. FHR is scored from zero to 6 for each hip and zero to 12 in total.

*Figure S2.* ***Three examples of progressively deteriorating Norberg Angle*** *The left is a radiograph of a hip joint showing a positive angle indicating good acetabular depth. The middle radiograph is an example of a small negative Norberg Angle, and the right radiograph an example of a large negative angle. Other signs of joint malformation and osteoarthritic effects may also be seen.* Images courtesy of Ruth Dennis
