# Supplementary figures and images for: Genetic Evaluation of the Nine Component Features of Hip Score in UK Labrador Retrievers
Source: PLoS One. 2010 Oct 22;5(10):e13610. doi: 10.1371/journal.pone.0013610 (PMC2962649; doi:10.1371/journal.pone.0013610)

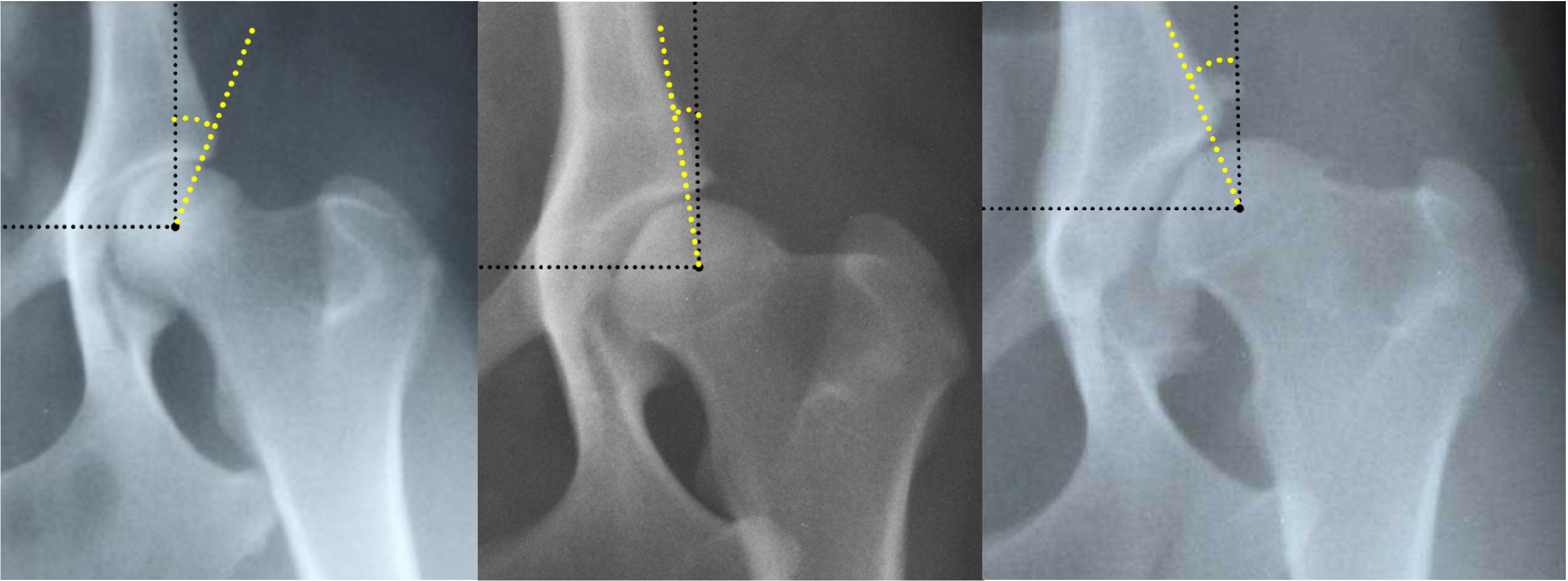

Supplement: Figure S1 — Three examples of progressively deteriorating Norberg Angle. The left is a radiograph of a hip joint showing a positive angle indicating good acetabular depth. The middle radiograph is an example of a small negative Norberg Angle, and the right radiograph an example of a large negative angle. Other signs of joint malformation and osteoarthritic effects may also be seen. Images courtesy of Ruth Dennis. (8.17 MB TIF) [file pone.0013610.s002.tif]
